# SILAR-Deposited CuO Nanostructured Films Doped with Zinc and Sodium for Improved CO_2_ Gas Detection

**DOI:** 10.3390/nano13202793

**Published:** 2023-10-19

**Authors:** Rana Saad, Ashour M. Ahmed, Khaled Abdelkarem, Mohamed Zayed, Zainab M. Faidey, Ghadah M. Al-Senani, Mohamed Shaban, Mohamed T. Tammam, Hany Hamdy

**Affiliations:** 1 Nanophotonics and Applications (NPA) Lab, Physics Department, Faculty of Science, Beni-Suef University, Beni-Suef 62514, Egyptoldfighter.khaled123@gmail.com (K.A.);; 2 Physics Department, College of Science, Imam Mohammad Ibn Saud Islamic University (IMSIU), Riyadh 11623, Saudi Arabia; 3 Department of Chemistry, College of Science, Princess Nourah bint Abdulrahman University, P.O. Box 84428, Riyadh 11671, Saudi Arabia; 4 Department of Physics, Faculty of Science, Islamic University of Madinah, P.O. Box 170, Madinah 42351, Saudi Arabia

**Keywords:** CuO/Zn thin films, CuO/Na thin films, gas sensing, SILAR, dynamic response, sensitivity, stability, selectivity

## Abstract

Gas sensing is of significant importance in a wide range of disciplines, including industrial safety and environmental monitoring. In this work, a low-cost SILAR (Successive Ionic Layer Adsorption and Reaction) technique was employed to fabricate pure CuO, Zn-doped CuO, and Na-doped CuO nanotextured films to efficiently detect CO_2_ gas. The structures, morphologies, chemical composition, and optical properties of all films are characterized using different tools. All films exhibit a crystalline monoclinic phase (tenorite) structure. The average crystallite size of pure CuO was 83.5 nm, whereas the values for CuO/Zn and CuO/Na were 73.15 nm and 63.08 nm, respectively. Subsequently, the gas-sensing capabilities of these films were evaluated for the detection of CO_2_ in terms of sensor response, selectivity, recovery time, response time, and limits of detection and quantification. The CuO/Na film offered the most pronounced sensitivity towards CO_2_ gas, as evidenced by a sensor response of 12.8% at room temperature and a low limit of detection (LoD) of 2.36 SCCM. The response of this sensor increased to 64.5% as the operating temperature increased to 150 °C. This study thus revealed a brand-new CuO/Na nanostructured film as a highly effective and economically viable sensor for the detection of CO_2_.

## 1. Introduction

Global warming and climate change represent pressing concerns as they result from the emission of greenhouse gases, such as carbon dioxide (CO_2_), into the atmosphere [1]. The consequent heat-trapping effect leads to unprecedented planetary warming, causing disruptions in weather patterns and ecological balance, thereby posing threats to diverse life forms [2]. Addressing this challenge necessitates monitoring and controlling CO_2_ levels in the atmosphere, sparking innovative research efforts [3]. The demand for cost-effective, highly sensitive gas sensors to achieve this goal continues to rise [4,5]. Various sensors have been devised to detect harmful gases, with semiconducting metal oxides (SMOs) emerging as excellent candidates, including CuO nanostructures [6,7,8,9]. CuO is a p-type semiconductor with unique properties due to its high copper or oxygen vacancies, safety, affordability, and excellent electrical conductivity [10,11]. Also, its features include a broad spectrum of light absorption, high melting temperatures, low cost, high hole mobility, good chemical stability, and a direct bandgap of 1.2–1.9 eV. This makes it promising for multiple applications, such as sensors, especially CO_2_ gas sensors [12,13,14,15], catalytic materials [16], and renewable energy devices [17,18,19]. Furthermore, researchers have looked into doping CuO with elements like Ba, Zn, Fe, Ti, and Ag to enhance its gas-sensing capabilities [20,21,22,23]. CuO@BaTiO_3_ nanocomposites with Ag were investigated by Lin et al. [24] for CO_2_ gas sensing, and a notable sensor response of 0.6% at 1000 ppm CO_2_ was obtained. A 6% Ba-doped CuO electrode operating at 30 °C was constructed utilizing the SILAR technique, and Khaled et al. [25] report that it was able to detect up to 9.4% of CO_2_ gas at a 100 SCCM flow rate with a recovery time of 5.44 s. CuO/ZnO bilayer thin film was developed and utilized by Bhowmick et al. [7] to achieve a 47% response for 2500 ppm CO_2_ at 375 °C. A CuO/CuFe_2_O_4_-based CO_2_ sensor was presented by Chapelle et al. [26]; it had an operating temperature of 250 °C, a response time of 55 min, and an 8 min recovery time at 5000 ppm. The sensor had a sensor response of 40%. Therefore, this study aims to design compact and low-cost nanostructured CuO-based sensors operating at low temperatures with high response, low response time, and recovery time beyond the previously reported values.

For CuO nanostructure fabrication, several methods are available, each with specific advantages depending on the intended application [27,28,29,30,31]. Among these, the successive ionic layer adsorption and reaction (SILAR) method, also known as solution-based atomic layer deposition (SALD), is notable for its cost-effectiveness, simplicity, and applicability for large-area deposition. However, this method has yet to be employed for the production of Na- or Zn-doped CuO films for room-temperature CO_2_ gas sensing. In this study, we used the SILAR approach to dope CuO thin films with Na and Zn to improve their CO_2_ gas sensing capabilities. Without post-annealing, we synthesized pure and doped films on glass substrates, tailoring the deposition conditions to produce adherent and high-quality films. Extensive analyses of chemical composition, structure, optical properties, and morphology were conducted. The study also explored the CO_2_ gas sensing performance, including dynamic response, sensor response, response time, recovery time, reusability, and selectivity, with the CuO/Na film exhibiting the most favorable performance.

## 2. Experimental Work

### 2.1. Materials

Copper chloride (CuCl_2_, molecular weight 170.48 g/mol, purity 99.5%) was supplied by Panreac, Spain. Sodium chloride (NaCl, 58.44 g/mol, 99.0% purity), zinc chloride (ZnCl_2_, 136.286 g/mol, 99.0% purity), and ammonium hydroxide (NH_4_OH, 35.04 g/mol, 30%) were supplied by Loba Chemie, India. All chemicals were used as received.

### 2.2. Synthesis of CuO Nanostructured Thin Films

Commercial glass slides were washed multiple times before deposition. The glass slides were cleaned using boiling diluted sulfuric acid, acetone, methanol, and deionized water (DIW) in an ultrasonic bath at room temperature for 20 min. Figure S1 (supplemental data) demonstrates four steps of the SILAR process, which involves immersing clean glass in cationic and anionic solutions. 0.1 M CuCl_2_ (250 mL) is mixed with NH_4_OH at pH 9 to form a cationic precursor. DIW served as an anionic precursor and was maintained at 90 °C. The cleaned glass substrate was soaked in a copper–ammonia mixture for 20 s to adsorb [Cu (NH_3_)_4_]^2+^ of the dark blue color. The glass substrate was washed into a DIW beaker for 30 s to transform the copper–ammonia complex [Cu(NH_3_)_4_]^2+^ into copper hydroxide (Cu(OH)_2_). The final stage involved immersing the Cu(OH)_2_-coated substrate in DIW at room temperature for 60 s under ultrasonic waves to remove weakly bound Cu(OH)_2_ molecules. To convert Cu(OH)_2_ to CuO thin film, the coated substrate was washed in DIW at 90 °C for 20 s. The CuO thin film was dried in the air for 10 s before the next cycle. This procedure is repeated forty times until a consistent dark brown coating covers the whole surface of the glass substrate. The chemical reaction that produces CuO can be identified using the following equations:

(1) $${\mathrm{CuCl}}_{2}+4\;{\mathrm{NH}}_{3}\cdot H_{2}O\to [\mathrm{Cu}({\mathrm{NH}}_{3}{)}_{4}{]}^{2+}+2\mathrm{HCl}$$

(2) $$[\mathrm{Cu}({\mathrm{NH}}_{3}{)}_{4}{]}^{2+}+4\;H_{2}O\;\to {Cu}^{2+}+4\;{\mathrm{NH}}_{4}+4\;{\mathrm{OH}}^{-}$$

(3) $${\mathrm{Cu}}^{2+}+2\;{\mathrm{OH}}^{-}\;\to \mathrm{Cu}(\mathrm{OH}{)}_{2}$$

(4) $$\mathrm{Cu}(\mathrm{OH}{)}_{2}\;\to \mathrm{CuO}\;(s)+H_{2}O$$

### 2.3. Synthesis of Na- and Zn-Doped CuO Nanostructured Thin Films

For enhanced CuO sensing properties, CuO was doped with 2% Na and 2% Zn by using the same method (SILAR). Sodium chloride (NaCl) and zinc acetate (Zn(CH_3_CO_2_)_2_) were used as sources for Na and Zn ions, respectively. These sources were mixed individually into a cationic precursor solution. Then the previous steps were repeated for the prepared CuO/Na and CuO/Zn films. All the thin films fabricated by the SILAR method were found to be stable with good adhesion to the glass substrate.

### 2.4. Nanostructured Thin Films Characterization

Surface morphologies of films were studied using a scanning electron microscope (SEM, JEOL, JSM-5400LV, Oxford, UK). The chemical compositions were studied using an energy dispersive X-ray (EDX) unit (JEOL JED-2300 SEM, Akishima City, Japan) with an accelerating voltage of 30 kV equipped with SEM. The crystal structure was examined using an X-ray diffractometer (XRD, PANalytical X’Pert Pro, Almelo, The Netherlands) with CuKα radiation in a scan range of 5 to 80°. The optical properties were scanned using a spectrophotometer (PerkinElmer Lambda 950, Waltham, MA, USA) ranging from 200 to 1200 nm with an increment of 1 nm.

### 2.5. Gas Sensing Measurements

**Figure 1:**
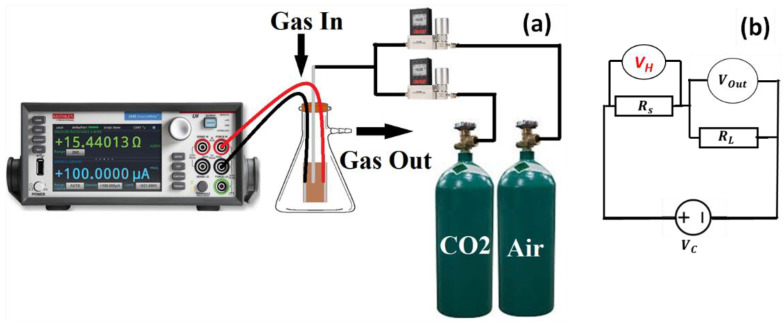
(**a**) Schematic diagram for the gas sensing measurement system and (**b**) metal oxide gas sensing circuit.

The gas sensing measurement system includes the fundamental measuring circuit for commercially available metal oxide gas sensors. A 1.0 L glass chamber with rubber O-rings on the top is prepared for gas sensing tests, as indicated in the schematic in Figure 1a. The chamber’s top surface features many holes: one for the gas inlet, one on the side for the gas outlet, and two for receiving electrical signals. CO_2_ gas and air are supplied via CO_2_ gas and air cylinders that are widely available. The flow rate of the gases is controlled using digital gas mass flow meters and controllers (Smart Track, Sierra Instruments, Monterey, CA, USA). To collect charges on the edges of thin films, conducting silver paste was added to both ends of the film, which functioned as electrodes. Keithley measurement-source unit (Tektronix 2450, Beaverton, OR, USA) was used for measuring the output electrical signal while changing the amount of CO_2_ that was put into the chamber. It should be mentioned that during data collection, the experimental equipment was kept at room temperature. As shown in Figure 1b, a load resistor (*R_L_*) is connected in series with a gas sensor in the measuring electric circuit for the gas sensor. The circuit voltage was set to *V_C_* = 1 V, and the output voltage (*V_out_*) equaled the load resistance voltage [32]. The sensor resistance (*R_S_*) was calculated from the measured value of *V_out_* using the relation $R_{s}=\frac{V_{C}-V_{out}}{V_{out}}.R_{L}$ [33]. The sensor’s working temperature was modified by adjusting the heating voltage (*V_H_*).

## 3. Results and Discussion

CuO thin film was doped with Na and Zn for structural, optical, and gas sensing improvements. The low-cost SILAR process makes pure and doped CuO films. Cu, Zn, and Na have different oxidation states, ionic radii, and electronegativity (Table S1) [34,35,36]. Cu^2+^ (0.072 nm) ions have a smaller ionic radius than Na^+^ (0.102 nm) and Zn^2+^ (0.074 nm). Zn^2+^ may have diffused more readily and affected the CuO crystallites because it has a smaller ionic radius than Na^+^. Changes in the ionic radius lead to variations in the positions of atoms within the crystal, which therefore result in dopant-induced structural changes in the CuO unit cell. According to the oxidation state, Cu^2+^ and Zn^2+^ have two positive charges, whereas Na^+^ has one. The oxidation state influences the carrier charge number. The Pauling scale shows that copper has a higher electronegativity (1.90) than zinc (1.65) and sodium (0.93). Higher electronegativity elements exhibit a stronger attraction to shared electrons. According to chemical processes, OH^−^ ions have a substantial effect on the growth of CuO films. Na^+^ and Zn^2+^ dopants in the precursor may consume OH^−^, affecting crystal formation. Based on the above, adding Na and Zn to CuO thin films should change their physical and chemical properties.

### 3.1. XRD Structural Analysis

**Figure 2:**
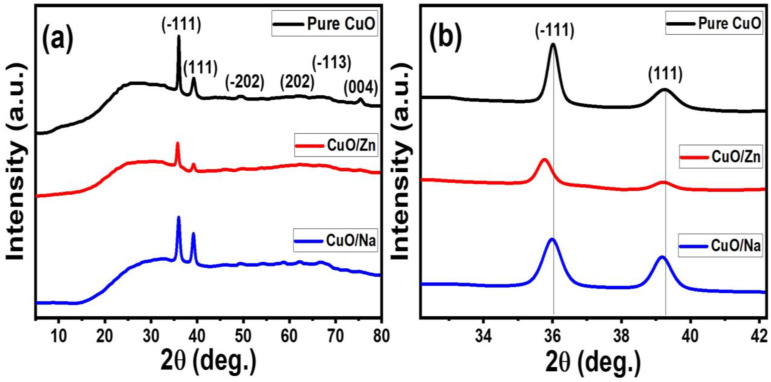
(**a**) XRD pattern for CuO, CuO/Zn, and CuO/Na and (**b**) peaks shift for (−111) and (111) planes.

XRD analysis was performed for all manufactured CuO thin films to determine whether Zn or Na dopants were integrated into the CuO crystal lattice by interstitial or substitutional processes. The XRD patterns of CuO, CuO/Zn, and CuO/Na films are shown in Figure 2. The XRD structural parameters for these films are illustrated in Table 1. If Na^+^ ions reside on Cu^2+^ sites, compensatory vacancies are generated in the O^2−^ sublattice due to impurities’ charges not matching hosts to maintain crystal electrical neutrality. The XRD patterns in Figure 2a reveal that all CuO films with and without doping exhibit crystalline monoclinic phase structure (tenorite) according to JCPDS card # 00-001-1117 with space group (C2/c). The crystallographic planes of (−111), (111), (−202), (202), (−113), and (004) for pure CuO films are located at 2θ-positions of 35.81°, 38.98°, 49.40°, 58.56°, 62.06°, and 75.44°, respectively [28]. In doped films, no further peaks related to Zn, Na, or any secondary phases were found. The impurity ions may be occupying vacant or interstitial sites in the CuO lattice crystal, which would explain why the peaks of Zn^2+^ and Na^+^ do not show up in XRD patterns. This suggests the successful incorporation of Zn^2+^ and Na^+^ ions into the CuO host lattice without affecting the crystal structure of the CuO [29,30].

The number of peaks decreased with doping. The intensities of the (−111) and (111) peaks are stronger than those of the other peaks. The intensity of the plane (−111) decreases after doping, indicating that the degree of crystallinity decreases for CuO/Zn and CuO/Na films. For CuO/Zn and CuO/Na, the positions of the main planes (−111) and (111) slightly shift compared to pure CuO, as seen in the XRD patterns in Figure 2b. This shift may be attributed to the lattice disorder in the CuO crystalline structure after doping. Similar results are obtained in previously reported works [37,38,39]. Vinay et al. reported that the shifting in the (−111) and (111) diffraction planes confirmed the distributions of Zn^2+^ ions in the CuO matrix [37]. Also, Haque et al. reported that there is a small shift in the XRD peak position of CuO/Na relative to pure CuO [40]. Wang et al. reported that the incorporation of Zn^2+^ ions into the CuO lattice can cause residual stress, which may lead to lattice deformation and, in turn, the occurrence of the left shift [41].

The crystallite size (D) for the two major peaks of the fabricated films was computed using the Debye–Scherrer equation, Equation (5) [42].

D = 0.94 λ/FWHM cos θ(5)

where λ is the wavelength of the incident X-ray (λ = 0.154 nm), FWHM is the full width at half maximum in radians, and θ is the diffraction angle.

The crystallite size was found to decrease and the FWHM increased with doping, as seen in Table 1. The average crystallite size for pure CuO was nearly 83.5 nm, and then it reduced to 73.15 nm for CuO/Zn and 63.08 nm for CuO/Na. This was attributed to the low ionic mobility and high radius of the Na ions in the solution.

The dislocation density (δ) is inversely proportional to the square of crystallite size, and its minimum value can be evaluated by using the following expression [43]:

(6) $$\delta =\frac{1}{D^{2}}$$

Doping with Zn and Na ions causes lattice deformation and defect growth, which raises the value of δ [38].

The microstrain (%) for (−111) and (111) of fabricated CuO, CuO/Zn, and CuO/Na thin films was calculated using Equation (7) [44,45].

(7) $$Microstrain\;\left(\%\right)=FWHM/4tan\;\theta$$

From Table 1, the CuO/Zn and CuO/Na films have more strain than the pure CuO film. This confirms the significant shift in the angle of diffraction and the peak intensity of both CuO/Zn and CuO/Na relative to pure CuO. The difference in the ionic radii causes a lattice strain and leads to changes in the lattice parameters. Also, consuming the OH^−^ ions by the dopants Na^+^ and Zn^2+^ affects the growth of the crystal structure.

The texture coefficient (TC) is an important parameter that indicates the preferred orientation of the growth of the synthesized nanocrystals. TC is estimated and listed in Table 1 by using the following equation [46]:

(8) $$TC\left(hkl\right)=\frac{I\left(hkl\right)/I_{0}\left(hkl\right)}{\left(N^{-1}\right)\left[\sum \frac{I\left(hkl\right)}{I_{0}\left(hkl\right)}\right]}$$

where I (hkl), I_0_ (hkl), and N are the measured plane intensity, the JCPDS standard intensity of a plane (hkl), and the number of diffraction peaks, respectively.

The intensity of the (−111) and (111) peaks for all CuO samples is much stronger than the other peaks. So, (−111) and (111) showed high TC values > 1, which indicates the favored orientation growth of the constructed nanocrystals along these directions (Table 1).

The interplanar distance (d-spacing) was calculated using Bragg’s formula [47]:

(9) m λ=2 sin θ

The interplanar spacing is inversely related to the angle of diffraction. The values of d-spacing increase after doping, as seen in Table 1. Expansions in the d-spacing of films indicate structural modification due to strain.

The lattice parameters of the monoclinic CuO thin films (a ≠ b ≠ c, α = γ = 90°, β ≠ 90°) and the volume of the unit cell (V) of the fabricated films are determined by using the following equations [37,48]:

(10) $$d\;(hkl)=\frac{1}{\sqrt{(\frac{h^{2}}{a^{2}\;{sin}^{2}(\beta )}+\frac{k^{2}}{b^{2}}+\frac{l^{2}}{l^{2}\;{sin}^{2}(\beta )}-\frac{2h\;l\;cos\;cos(\beta )}{a\;c\;{sin}^{2}(\beta )}}}$$

(11) V=a b c sin (β)

where β is the angle between the a and b axes (β ≠ 90°).

Lattice parameters and the volume of the unit cell for pure CuO are obtained as a = 4.618 Å, b = 3.126 Å, c = 5.059 Å, and V = 79.89 Å^3^. These results are well matched with reference JCPDS card # 00-001-1117. For CuO/Zn and CuO/Na, the volume of the unit cell slightly decreases due to the volume being proportional to the lattice parameter.

The specific surface area (SSA) is a favorable parameter for liquid diffusion and gas adsorption. The SSA can be calculated using the crystallite size (D) and the density of the film (ρ) according to Equation (12).

(12) $$SSA\left(m^{2}g^{-1}\right)=6000/D\rho$$

The calculated SSA value for CuO/Na (1.492 $m^{2}g^{-1}$) film is higher than that calculated for CuO (1.137 $m^{2}g^{-1}$) and CuO/Zn (1.290 $m^{2}g^{-1}$) films, as seen in Table 1.

### 3.2. Surface Analysis and Roughness Parameters

**Figure 3:**
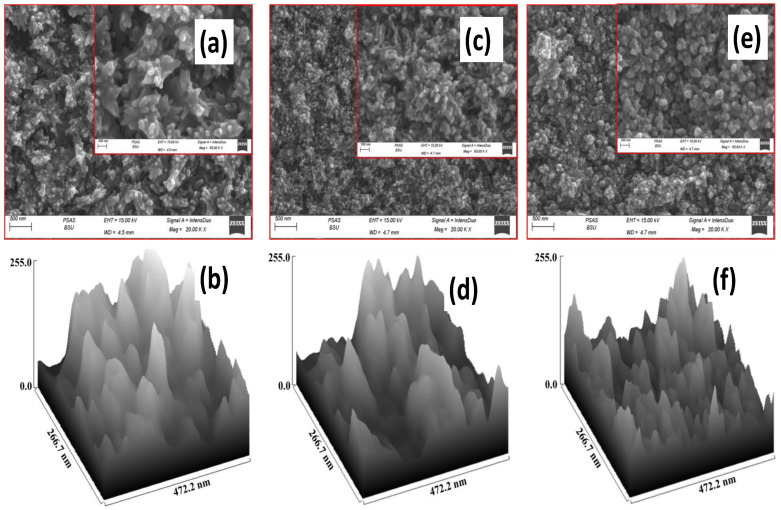
SEM images and surface morphology for CuO (**a**,**b**), CuO/Zn (**c**,**d**), and CuO/Na (**e**,**f**) films.

The surface morphologies of the produced films were studied using SEM, a crucial technique that provides particle shape and size information. CuO, CuO/Zn, and CuO/Na SEM micrographs are shown in Figure 3. The SEM image was analyzed using the ImageJ software (version: 1.53e). According to Figure 3a,c,e, all films showed nanoparticulate surfaces of agglomerated nanoparticles. These agglomerations are randomly distributed to continuously cover the glass substrates. Because of the low growing temperature, no voids or cracks are observed on the films’ surfaces. Particles tend to cluster and agglomerate, resulting in rough surfaces that are closely packed. The surface morphology of pure CuO nanostructures is different from that of CuO/Zn and CuO/Na films, as shown in Figure 3c,e. On the other hand, the particles become denser and smaller to form aggregations after adding Na and Zn to CuO films. This alteration may be attributed to the difference in ionic radii between dopant elements and consuming OH^−^ ions in the solution, which affects the growth of the crystal structure and the morphology [49,50,51].

Surface roughness facilitates the analysis of chemical reactions and gas adsorption on film surfaces. The SEM images were analyzed using Gwyddion software (Version: 2.63). This software provides open-source data for processing and statistical analysis of SEM images. It accurately measures roughness parameters. Figure 3b,d,f shows CuO, CuO/Zn, and CuO/Na rough surfaces. The surface development of films revealed dips and peaks. Compared to pure CuO, CuO/Zn and CuO/Na have rougher surfaces. Reduced particle size could result in more surface roughness [52]. It is noticed that the CuO/Na film has the highest surface roughness and lower particle size, indicating a high surface area, which is very useful in the CO_2_ gas sensor application.

The finer and non-periodic irregularities in the surface texture are measured by the roughness parameters. Surface roughness can be characterized using a wide range of factors. Arithmetic average roughness (R_a_) is the average surface roughness over the whole length of the measurement. It shows the average valley–peak disparity. Height variance is often measured using root mean square roughness (RMS or R_q_). After squaring each height value in the dataset, the square root of the mean is used to calculate it. Due to its resistance to scratches, impurities, and measurement noise, the parameter is easy to statistically treat and produces trustworthy findings. Maximum roughness (R_t_) is the peak height plus valley depth. Despite its extensive use, maximum height is subject to scratches, pollution, and measuring noise because it relies on peak values. Maximum roughness (R_tm_) is the average of the highest peak height and maximum valley depth of Skewness (R_sk_). This metric measures height dispersion. R_SK_ = 0: equilateral to the average line (normal distribution); R_SK_ > 0: standard deviation greater than zero; R_SK_ < 0: standard deviation less than zero. Kurtosis (R_ku_), which is connected to the geometry of peaks and valleys at their points, can be used to assess contact between two objects. R_ku_ = 3 in a typical case; R_ku_ > 3 indicates a height discontinuity, whereas R_ku_ < 3 indicates a uniform height distribution. The values of R_a_, R_q_, R_t_, R_tm_, R_sk_, and R_ku_ for all films are shown in Table 2. The CuO/Na film has higher roughness metrics than the others. This implies that CuO/Na is better for gas detection.

From the SEM image, the particle size distribution histograms are shown in Figure S2a–c (supplementary data). The average particle size for pure CuO is 91.6 nm, and the particle size ranges from 60 to 120 nm. For CuO/Zn and CuO/Na, the average particle size dropped to 63.5 nm and 51.27 nm, respectively, Figure S2d. The effective incorporation of Na and Zn into the CuO lattice may be the cause of the reduction in particle size.

### 3.3. Energy Dispersive X-ray Spectroscopy Analysis (EDX)

**Figure 4:**
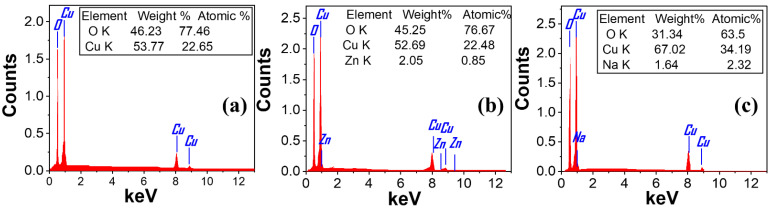
EDX spectrum of (**a**) pure CuO, (**b**) CuO/Zn, and (**c**) CuO/Na thin films.

Energy-dispersive X-ray spectroscopy (EDX) was used to analyze the chemical composition of the produced films and validate the effective incorporation of Zn and Na into the CuO nanostructure. Figure 4 shows the EDX spectra of pure CuO, CuO/Zn, and CuO/Na. The quantitative data of the EDX analysis is in the inset tables. The EDX chart shows the existence of both Cu and O elements for all thin films, as shown in Figure 4a. There were no extra peaks observed, thereby confirming the high purity of synthesized CuO as illustrated in the XRD analysis. From Figure 4b,c, the peaks for Zn and Na appeared in the EDX for CuO/Zn and CuO/Na films, respectively. Pure CuO contains 53.77% Cu and 46.23% O by weight ratio. The weight ratio for Cu and O slightly changes in the CuO/Zn film, whereas there is a high change in the CuO/Na film. This may be the difference between the valance state, ionic radius, and electronegativity for Na^+^ and Cu^2+^ ions.

### 3.4. Optical Analysis

**Figure 5:**
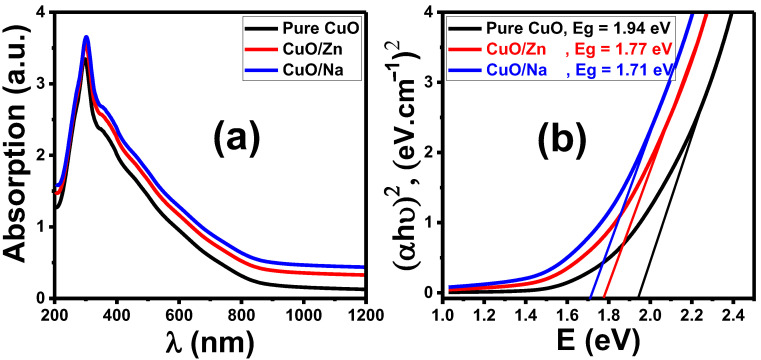
(**a**) Optical absorbance spectra and (**b**) optical bandgap for pure CuO, CuO/Na, CuO/Zn films.

Optical absorption studies were performed using a UV-Vis spectrophotometer over the wavelength ranging from 200 to 1200 nm to clarify the optical bandgap. The optical properties of thin films depend on several factors, like surface morphology, particle size, doping element, and micro-strain [53]. Figure 5 shows the absorbance and bandgap spectra of CuO, CuO/Na, and CuO/Zn nanostructured films. The absorbance values of the fabricated films are significantly affected by the Zn and Na doping of CuO. From Figure 5a, all films show a strong absorption band in the UV region (nearly 300 nm), resulting in an electron jump from the valence band to the conduction band of CuO. Absorption gradually decreased in the visible region and remained constant above 800 nm. CuO/Na samples show higher absorbance values than pure CuO and CuO/Zn films. Compared to pure CuO nanostructured thin film, the absorption band edge of CuO/Zn and CuO/Na slightly redshifted towards higher wavelengths, which indicates a narrow band gap. The absorbance edge of CuO/Zn and CuO/Na slightly shifted towards a longer wavelength (red shift) compared to undoped CuO in many previous works [54].

For the direct bandgap transition, the energy bandgap can be assessed using the Tauc equation, Equation (13), as plotted in Figure 5b.

(13) $${\left(\alpha \;E_{ph}\right)}^{2}=X\;(E_{ph}-Eg{)}^{}$$

where α is the absorption coefficient, E_ph_ is the energy of an incident photon, Eg is the energy of the bandgap, and X is the independent energy constant. The Eg can be estimated from the intercept of the plots of (α E_ph_)^2^ versus (hν) (where Eg = E_ph_ when α = 0). As shown in Figure 5b, the band gaps of pure CuO, CuO/Zn, and CuO/Na are 1.94 eV, 1.77 eV, and 1.71 eV, respectively. In general, the addition of dopant impurities can cause a reduction in particle size, an increase in surface roughness, and a decrease in crystallinity, which can result in scattering of the incident light and an increase in optical absorption. The presence of impurity atoms can act as an activator for the development of localized energy levels in forbidden energy gaps that extend into the bandgap below the conduction band, resulting in a reduction in the optical band gap in such materials [53,54].

The incorporation of Zn into CuO nanostructures has resulted in improvements in the absorption of light and a reduction in Eg. This may be attributed to the d-d transition between closely spaced Zn^2+^ and Cu^2+^ ions and the combined transition from oxygen 2p states to d-sates of Cu and Zn [53,55]. Using Na^+^ ions rather than Cu^2+^ ions can change the charge density and cause a shift in the Fermi level accompanied by changes in the energy gap of CuO, which makes it possible to absorb a photon with a lower energy level in the visible region [56,57]. The decrease in the optical energy gap (bandgap) of CuO/Zn and CuO/Na thin films can be attributed to the influence of Zn and Na doping, which introduces new energy states within the bandgap, affecting the electronic structure and optical properties of the materials. When CuO is doped with Zn or Na, the introduced dopant atoms substitute some of the Cu atoms in the CuO lattice, creating localized energy states within the bandgap. These dopant atoms have different electron configurations compared to Cu atoms, and this leads to alterations in the electronic structure and the band structure of the material. Zn is known as a shallow acceptor dopant in the CuO lattice. Shallow acceptors create acceptor levels near the valence band edge, resulting in the introduction of energy levels within the bandgap. These energy levels effectively reduce the bandgap, as electrons can easily transition from the valence band to these acceptor levels, requiring less energy [57,58,59,60,61]. Na-doping in CuO is likely to create similar localized states within the bandgap. Na can act as a donor or acceptor dopant depending on the local conditions, but in this case, it may act as an acceptor or create shallow acceptor levels, which could also lead to a decrease in the bandgap [40,62]. The decrease in the bandgap indicates that these materials require less energy to promote electrons from the valence band to the conduction band, making them more sensitive to electromagnetic radiation in the visible spectrum. This effect has a significant impact on the optical properties and potential applications of these materials in optoelectronic devices, including sensors and photodetectors.

The band gap is a very important property of a semiconductor because it determines its electrical conductivity [63]. This is because the gas sensor depends on changes in the conductivity of the CuO film during the passage of gas molecules. It is expected that the CuO/Na film is more suitable for sensing applications.

The refractive index is an important parameter to characterize the electronic and optical properties of semiconductors. The relation between the optical energy gap and the static refractive index of semiconductors may be described by Equation (14) [64].

(14) $$n=\sqrt{(\frac{11.8336}{E_{g}^{0.5}})-1.85472}$$

The refractive indexes of CuO, CuO/Zn, and CuO/Na are 2.57, 2.65, and 2.68, respectively.

### 3.5. I-V Characteristic Curve

**Figure 6:**
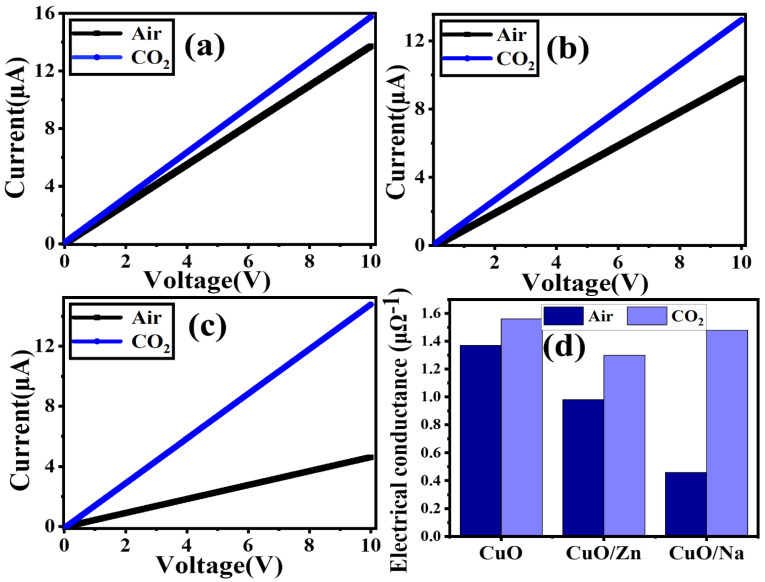
I-V curves of (**a**) pure CuO, (**b**) CuO/Zn, and (**c**) CuO/Na at 50 SCCM CO_2_ gas and air at room temperature, and (**d**) the electrical conductance of thin films in Air and CO_2_.

Figure 6a–c depicts their current–voltage (I-V) characteristics throughout a voltage range of 0 V to 10 V at room temperature for all films. The I-V curve was measured in air and CO_2_ gas at 50 SCCM. All films exhibit good linear I-V characteristics. An increase in DC applied voltage leads to an increase in the current value. This indicates that CuO thin films make ohmic electrical contact with the electrodes. It is related to the sensing material’s transmission of charge carriers [65]. CO_2_ is a non-polar oxidizing gas. The CuO exhibited p-type behavior, implying that p-type materials conduct using positive holes rather than electrons as the majority charge carrier. When exposed to varying amounts of CO_2_ gas, the resistance of the CuO sensor is reduced, and the current of the CuO sensor rises [7]. This indicates increased conductance in the presence of CO_2_ gas. At 50 SCCM, the conductance is increased from 1.37 to 1.56 μΩ^−1^ for pure CuO, from 0.98 to 1.3 μΩ^−1^ for CuO/Zn, and from 0.46 to 1.48 μΩ^−1^ for CuO/Na, as shown in Figure 6d. Another important observation from Figure 6b is that the conductance of CuO/Na increases three times after passing CO_2_ gas. Previous works showed that doping CuO films with Na and Zn enhanced their conductivity [7]. The electrical conductance of a material depends on its overall characteristics, such as crystal structure, surface morphology, chemical composition, type of doping, surface roughness, and charge carrier concentration.

### 3.6. Dynamic Sensing Response to CO_2_ Gas

**Figure 7:**
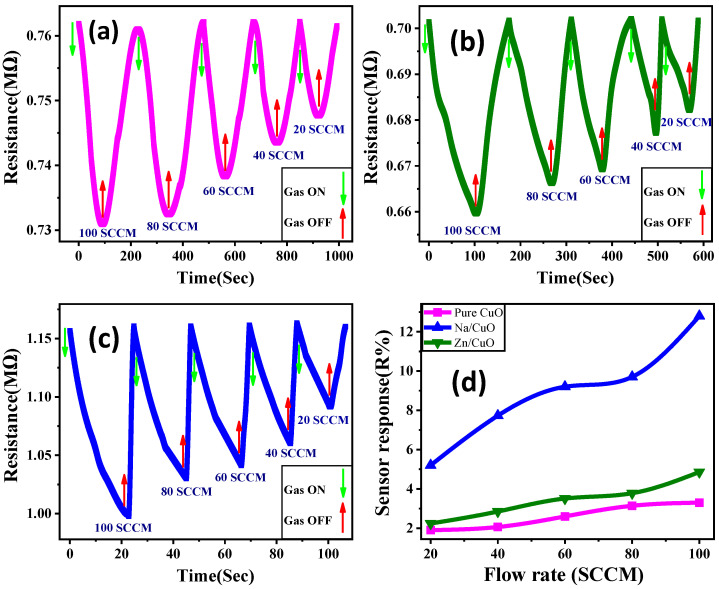
Response behavior of the CuO sensors: (**a**) CuO pure, (**b**) CuO/Zn, and (**c**) CuO/Na, and (**d**) the sensor response of CuO thin films at different gas flow rates.

The sensitive layers’ resistance was tested under different CO_2_ gas flow rates in the air at room temperature. Figure 7 shows response and recovery characteristics. This figure shows sensor response and gas flow rate for pure CuO, CuO/Zn, and CuO/Na. Measurements were carried out at 20–100 SCCM intervals. We examined how CuO thin films react to CO_2_, how stable they are, and how rapidly they recover from gas reactions. CO_2_ gas interacts with CuO sensor surfaces, changing charge carrier concentration and sensor resistance. Pumping CO_2_ into CuO sensors reduced their resistance. After gas discharge, the sensors’ resistance returned to its normal value. Figure 7a–c shows that at a 100 SCCM flow rate, the resistance of CuO/Na is significantly lower than that of CuO/Zn and pure CuO, and the sensor response increases with doping. Hence, as shown in Figure 7d, the CuO/Na films exhibit superior performance when compared to the CuO and CuO/Zn films. Based on the dynamic response in Figure 7a–c, the sensor response (R%) can be calculated using Equation (15).

(15) $$R\%=\left|\frac{R_{{CO}_{2}}-R_{air}}{R_{air}}\right|\times 100$$

Here $R_{CO_{2}}$ and $R_{air}$ refer to the measured resistance in CO_2_ and air environments, respectively. The value of $R_{CO_{2}}$ is obtained from the dynamic response after a certain exposure time to CO_2_, and the value of $R_{air}$ is measured under the same conditions [66]. As shown in Figure 7d, doping CuO thin film enhances sensor responsiveness, especially with Na. As the flow rate increases from 20 SCCM to 100 SCCM, the pure CuO sensor response increases from 1.9% to 3.3%. On the other hand, the sensor response for CuO/Zn rises from 2.24% to 4.86%, and the CuO/Na sensor response increases from 5.2% to 12.8%.

### 3.7. Response, and Recovery Time

**Figure 8:**
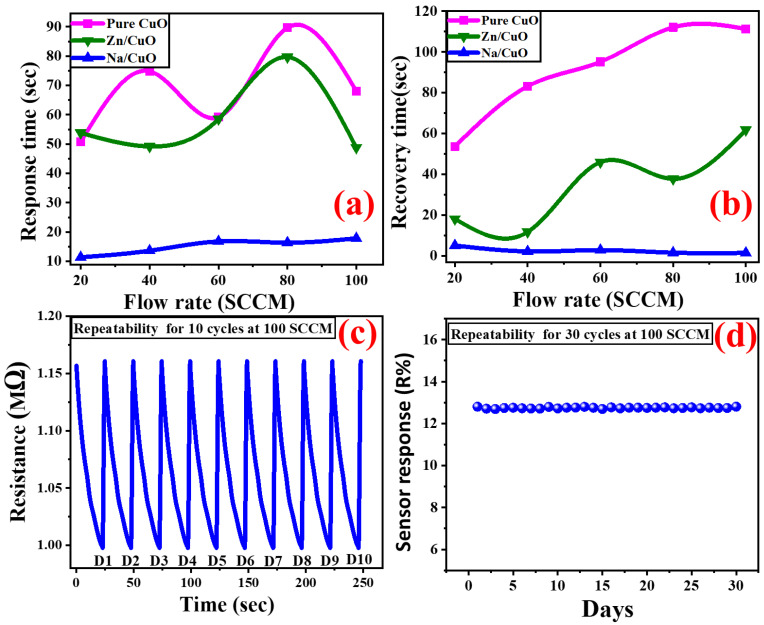
(**a**) The response time and (**b**) the recovery time of pure and doped CuO thin films versus different gas flow rates; the repeatability of the CuO/Na when exposed to 100 SCCM flow rate of CO_2_ at room temperature in terms of (**c**) sensor resistance and (**d**) sensor response (R%).

According to Figure 7d and Figure 8a,b, CuO/Na is the most sensitive sensor to CO_2_ gas and has the lowest response and recovery time due to its high surface roughness and low particle size [67,68]. The period following CO_2_ injection that elapses before the relative resistance reaches 90% of the steady-state value is known as the response time. The estimated CuO sensor response time versus the gas flow rate is shown in Figure 8a. Recovery time is the time taken by the sensor to reach a resistance 10% above the original value. Figure 8b shows the predicted recovery times versus the flow rate. At 100 SCCM CO_2_, CuO/Na responds in 17.84 s, CuO/Zn in 48.8 s, and pure CuO in 68.06 s. CuO/Na recovers in 1.5 s, CuO/Zn in 61.83 s, and pure CuO in 111.32 s; i.e., doping reduces response and recovery times, whereas Na is more preferred than Zn as a dopant. Low particle size and strong surface roughness improve CO_2_ gas responsiveness and recovery in the film [66]. The CuO/Na has the quickest recovery time when compared to other CuO-based CO_2_ sensors that have been previously reported in the literature [7,24,25,69,70,71,72,73,74,75]. The addition of Na led to improved gas adsorption and reaction kinetics; it also reduced the size of the nanoparticles and raised the surface area-to-volume ratio. Na dopant can also alter the electronic properties of CuO, which could result in faster reaction/recovery times and enhanced sensor response. When Na or Zn is combined with CuO in the nanostructured film, a synergistic effect that increases the characteristics of both materials can be produced; this could lead to improved gas sensing performance when compared to pure CuO or alternatives that do not exhibit the same synergistic effect.

### 3.8. Limit of Detection and Limit of Quantification

The limit of detection and the limit of quantification are very important factors in evaluating the sensor’s performance. The limit of detection (LoD) is the lowest value of the flow rate of CO_2_ gas that can be reliably detected by the designed sensor. The LoD is calculated from the linear calibration curve at low concentrations using the standard deviation (SD) and the slope of the curve. A very low LoD shows a good sensor that is capable of resolving very small changes in the flow rate. It can be expressed by using Equation (16).

(16) $$LoD=3\times \frac{Standard\;deviation}{Slope}$$

The limit of quantification (QoL) is the lowest amount of CO_2_ molecules that can be measured with acceptable accuracy and precision. We were able to determine the sensor’s limit of quantification using Equation (17).

(17) $$QoL=10\times \frac{Standard\;deviation}{Slope}$$

The signal-to-noise ratio (SNR) is used to compare the level of the desired signal to the level of background noise. It is defined as the ratio of desired output signal power to noise power (unwanted signal), as shown in Equation (18).

(18) $$\frac{S}{N}=\frac{2H}{h}$$

where H is the height of the peak and h is the FWHM at low concentration. Also, it is equal to the ratio of the mean to the standard deviation of the measurement. The higher the SNR, the better the signal quality. The values of SD, slope, LoD, QoL, and SNR are summarized in Table 3 for all films. The LoD for pure CuO, CuO/Zn, and CuO/Na are calculated to be 17.9 SCCM, 15.9 SCCM, and 2.36 SCCM, respectively. After doping, the slope and SD values rise. The best sensor, as determined using LoD values, is CuO/Na, which has the lowest detection limit [76]. CuO/Na also has the best rating for QoL.

### 3.9. Reducibility and Repeatability

For practical applications, another effective factor of a gas sensor is how stable it is over time. Figure 8c,d shows how the CuO/Na sensor changed after being exposed to 100 SCCM CO_2_ for 30 days at RT. This figure shows that the initial resistance of the sensor in the air stayed almost the same during the 30 days of continuous testing. This showed that the materials used to manufacture the sensor were very stable. During the test, the sensor’s response value stayed around 12.8%. This showed that the sensor is stable and has good repeatability. The good repeatability of the CuO/Na sensor changed after being exposed to 100 SCCM NH_3_, and CH_3_OH for 30 days at RT, as shown in Figure S3 (supplementary data).

### 3.10. Selectivity and Effect of Temperature

When establishing the features of gas sensors, selectivity is a crucial factor to keep in mind. Metal oxide-based gas sensors struggle significantly with selectivity. Most of the time, the way to test sensor selectivity is to expose the device being tested to different gases at different times and measure the sensor response ratio for each gas, as shown in Equation (19).

(19) $$Selectivity(\eta \%)=\frac{R_{other\;gas}}{R_{target\;gas}}\times 100$$

**Figure 9:**
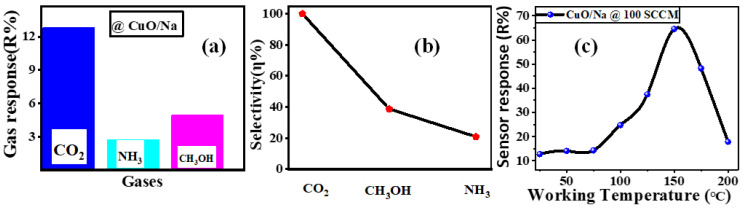
(**a**) Response and (**b**) selectivity of the CuO/Na sensor for various gases at 100 SCCM flow rate and room temperature, and (**c**) effect of temperature on the response of CuO/Na thin film at a flow rate of 100 SCCM CO_2_.

So, the sensor response of the CuO/Na thin film was tested by exposing it to CO_2_, NH_3_, and CH_3_OH at a flow rate of 100 SCCM at room temperature. Figure 9a shows that the sensor’s response was higher to CO_2_ than to the other gases tested (R_CO2_ > R_CH3OH_ > R_NH3_). The selectivity study for various gases relative to CO_2_ is shown in Figure 9b. Its percentages are 38.8% and 20.9% for CH_3_OH, and NH_3_, respectively. The higher sensor response towards CO_2_ than CH_3_OH and HN_3_ can be explained based on different interactions between sensing film and adsorbed gas. The interaction of CH_3_OH and HN_3_ with CuO film is very weak compared to CO_2_, hence it shows less response to these gases.

The improvement in the responsiveness of the CuO/Na sensor was noticed when the operating temperature was increased from 25 to 150 °C, as depicted in Figure 9c. After these enhancements, decreases in performance were observed as the operational temperature increased to 200 °C. The sensor response value of CuO/Na at 150 °C was measured to be 64.5%, which was nearly five times higher than the value seen at room temperature (RT). This finding suggests that the optimal working temperature range for CuO/Na lies within 150 ± 5 °C. However, our objective is to operate at ambient temperature to enhance use and accessibility. This sensing element showed a higher response at lower operating temperatures when compared to the CuO-based CO_2_ sensors that were previously reported [7,24,69].

### 3.11. Gas Sensing Mechanism

**Figure 10:**
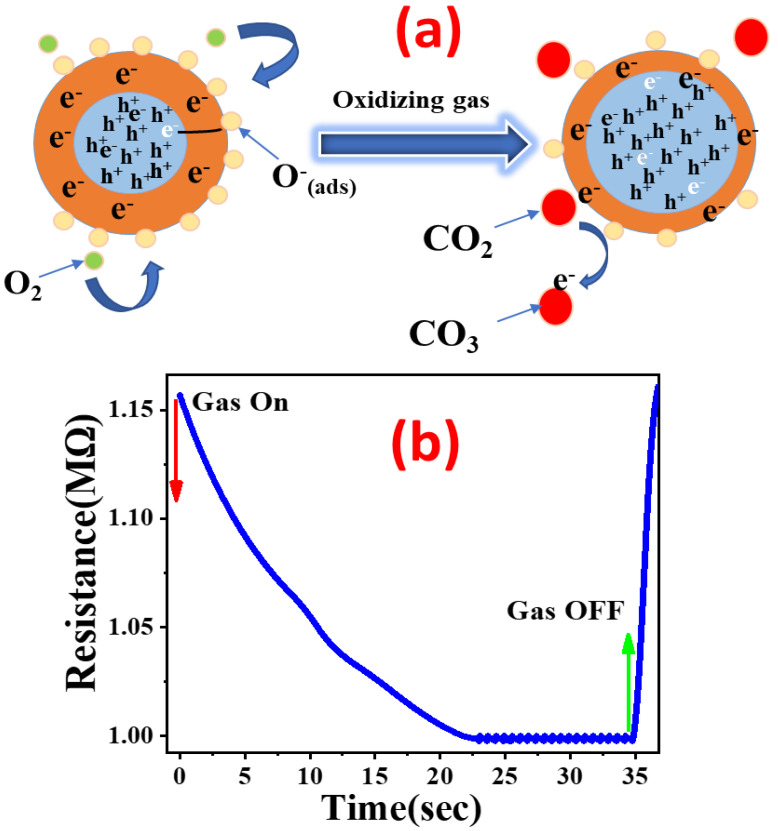
The gas-sensing mechanism (**a**) and related resistance variation (**b**).

Many authors have previously described the adsorption of oxidizing gases on defect sites of metal oxide semiconductors [32,77,78]. Figure 10a,b shows the gas sensing mechanism and resistance variation. It is generally agreed that the p-type behavior of CuO can be attributed to a copper vacancy, V″_Cu_, as shown in Figure 10a. When exposed to gaseous oxygen in the air, the CuO sensing element adsorbs the oxygen at the copper vacancies using the following formula:

(20) $$V_{Cu}^{\prime \prime }+\frac{1}{2}O_{2}\to V_{Cu}^{\prime }+O_{\left(ads.\right)}^{-}+h_{}^{+}$$

After oxygen adsorption, a layer of hole accumulation is formed on the CuO surface, which causes a reduction in its resistance. CO_2_ acts as an oxidizing gas during its contact with CuO. When CO_2_ adsorbs on all possible CuO defect sites by absorbing electrons, resulting in a further reduction in resistance.

(21) $$V_{Cu}^{\prime \prime }+{CO}_{2(gas)}\to V_{Cu}^{\prime }+{CO}_{2(ads.)}^{-}+h_{}^{+}$$

After the CO_2_ adsorption process is complete, carbonates are produced when the CO_2_ reacts with the adsorbed oxygen.

(22) $${CO}_{2(ads.)}^{-}+O_{\left(ads.\right)}^{-}\to {CO}_{3(gas)}$$

The resulting carbonates even reduce the resistance, as shown in Figure 10b. Carbonates are generated during response breakdown into CO_2_ and O_2_ after recovery, which increases CuO resistance to return to its stable value [7].

Finally, Table 4 provides a comparison of the performance of several nanomaterials for sensing CO_2_ gas in the present work and previous studies [7,25,26,69,70,71,72,73,74,75]. The response of the suggested sensor is higher than that of many values for the sensors previously reported in the literature, as shown in Table 4. This indicates that our optimized sensor can be used as a starting point for the design of more efficient CO_2_ sensors at room temperature using the low-cost SILAR technique. The higher CO_2_ gas sensor response, lower response time, and recovery time observed in Na/CuO nanostructured film can be attributed to the enhanced surface area, improved sensitivity, catalytic activity, and synergistic effect of the materials [7,25,26,69,70,71,72,73,74,75].

## 4. Conclusions

Pure CuO, Zn-doped CuO, and Na-doped CuO films were synthesized via the low-cost SILAR process for CO_2_ sensing. Zn and Na doping effects on the structural, optical, and gas-sensing attributes of CuO films were investigated. Notably, the CuO/Na film displayed the smallest crystallite size and the highest surface roughness compared to pure CuO and CuO/Zn films. The CO_2_ sensing performance and mechanism were explored, revealing that the CuO/Na film exhibited superior CO_2_ detection capabilities (R% = 12.8%, DoL = 2.36 SCCM, S/N = 2.29) at room temperature. Furthermore, this film demonstrated remarkable stability and selectivity for CO_2_ detection, with a 17.84 s response time at 100 SCCM CO_2_ and a rapid 1.5 s recovery time for CuO/Na. In contrast, CuO/Zn and pure CuO exhibited longer response and recovery times (48.8 s and 61.83 s for CuO/Zn, 68.06 s and 111.32 s for pure CuO). As the operational temperature rose to 150 °C, the sensor’s response climbed to 64.5% or nearly five times its performance at room temperature. This study presents new opportunities for CuO/Na nanostructured design and CO_2_ sensing performance when compared to previously reported CuO-based sensors because of the low-cost design, better responsiveness, lower response time, recovery time, and detection limit.

## Figures and Tables

**Table 1:** The XRD parameters for the CuO, CuO/Zn, and CuO/Na thin films for (−111) and (111) planes.

XRD Parameters	Pure CuO		CuO/Zn		CuO/Na	
	(−111)	(111)	(−111)	(111)	(−111)	(111)
2θ (°)	35.99	38.22	35.74	39.18	35.96	39.15
d-value (Å)	2.495	2.297	2.512	2.299	2.498	2.301
Rel. Intensity (%)	100	31.76	100	30.08	100	68.67
Measured Intensity	339.63	107.86	140.19	42.17	263.59	181.01
FWHM (°)	0.354	0.787	0.472	0.629	0.629	0.629
TC	1.52	0.48	1.54	0.46	1.19	0.81
D (nm)	83.27	83.82	73.22	74.07	63.27	64.06
δ (nm^−2^) × 10^−5^	14.42	14.23	18.65	18.22	24.98	24.36
Microstrain (%) × 10^−3^	4.76	5.91	6.39	7.72	8.47	7.73
a (Å)	4.618		4.525		4.499	
b (Å)	3.126		3.098		3.012	
c (Å)	5.059		5.011		5.001	
V (Å^3^)	79.89		77.91		76.82	
SSA $\left(m^{2}g^{-1}\right)$	1.137		1.290		1.492	

**Table 2:** Roughness parameters for all thin films.

Parameters	Pure CuO	CuO/Zn	CuO/Na
Arithmetic average roughness (R_a_) (μm)	0.833	2.164	2.65
Root mean square roughness (R_q_) (μm)	1.047	2.576	3.35
Maximum height of roughness (R_t_) (μm)	5.34	12.45	19.66
Average Maximum height of roughness (R_tm_) (μm)	4.11	8.685	14.82
Skewness (R_sk_)	0.05	−0.0558	0.12
Kurtosis (R_ku_)	2.5	2.688	3.29

**Table 3:** Values of SD, slope, LoD, QoL, and SNR parameters.

	CuO	CuO/Zn	CuO/Na
Slope (Ω/s)	240.14	302.07	4704.73
SD	1432.25	1607.77	3707.69
LoD (SCCM)	17.9	15.9	2.36
QoL	59.64	53.22	7.88
H	11.454	12.045	11.582
h	9.49	9.94	10.10
SNR	2.41	2.68	2.29

**Table 4:** Comparison of the performance of several nanomaterials for sensing the CO_2_ gas with our optimized sensor.

Nanostructured	Temperature (°C)	R (%)	t_response_ (s)	t_recovery_ (s)	Ref.
CuO-ZnO (C/Z)	375	47	44 s	217 s	[7]
CuO: 6% Ba	RT	9.4	5.6 s	5.44 s	[25]
CuO/CuFe_2_O_4_	250	40	55 min	8 min	[26]
Porous LaFeO_3_/SnO_2_ nanocomposite film	250	2.75	18 s	--	[69]
La_0.8_Sr_0.2_FeO_3_	380	1.25	660	300	[70]
BaTiO_3_-CuO	300	9	120	120	[71]
ZnO nanowire	200	3.8	8	40	[72]
LaOCl-coated ZnO NWs	400	4.0	18	12	[73]
LaBaCo_2_O_5+δ_	300	4	--	--	[74]
CuO	RT	3.5	10 s	6 s	[75]
CuO/Na	25	12.8	17.48	1.5	This work
CuO/Na	150	64.5			This work

## Data Availability

Not applicable.

## Supplementary Materials

The following supporting information can be downloaded at: https://www.mdpi.com/article/10.3390/nano13202793/s1, Figure S1: Schematic representation of the used modified SILAR technique to produce pure and doped CuO films; Figure S2: The particle size histogram for (a) CuO, (b) CuO/Zn, and (c) CuO/Na films; Figure S3: The repeatability of the CuO/Na when exposed to 100 SCCM flow rate of (a,b) CH3OH and (c,d) NH_3_ at room temperature in terms of (a,c) sensor resistance and (b,d) sensor response (R%); Table S1: The oxidation state, ionic radius, and electronegativity for Cu, Zn, and Na.
