# Supplementary material for: SILAR-Deposited CuO Nanostructured Films Doped with Zinc and Sodium for Improved CO2 Gas Detection
Source: Nanomaterials (Basel). 2023 Oct 19;13(20):2793. doi: 10.3390/nano13202793 (PMC10609130; doi:10.3390/nano13202793)
Supplement: Supplementary file 1 [file nanomaterials-13-02793-s001.zip › nanomaterials-2651887-supplementary.pdf]

Supplementary data

SILAR-Deposited CuO Nanostructured Films Doped with Zinc and Sodium for Improved CO_2 Gas Detection

Rana Saad[1], Ashour M. Ahmed[1,2], Khaled Abdelkarem[1], Mohamed Zayed[1], Zainab M. Faidey[1], Ghadah M. Al-Senani[3], Mohamed Shaban[4]*, Mohamed T. Tammam[1], Hany Hamdy[1]

[1] Nanophotonics and Applications (NPA) Lab, Physics Department, Faculty of Science, Beni-Suef University, Beni-Suef 62514, Egypt
[2] Physics Department, College of Science, Imam Mohammad Ibn Saud Islamic University (IMSIU), Riyadh 11623, Saudi Arabia
[3] Department of Chemistry, College of Science, Princess Nourah bint Abdulrahman University, P.O. Box 84428, Riyadh 11671, Saudi Arabia
[4] Department of Physics, Faculty of Science, Islamic University of Madinah, P.O. Box 170, Madinah 42351, Saudi Arabia
* Correspondence: mssfadel@aucegypt.edu

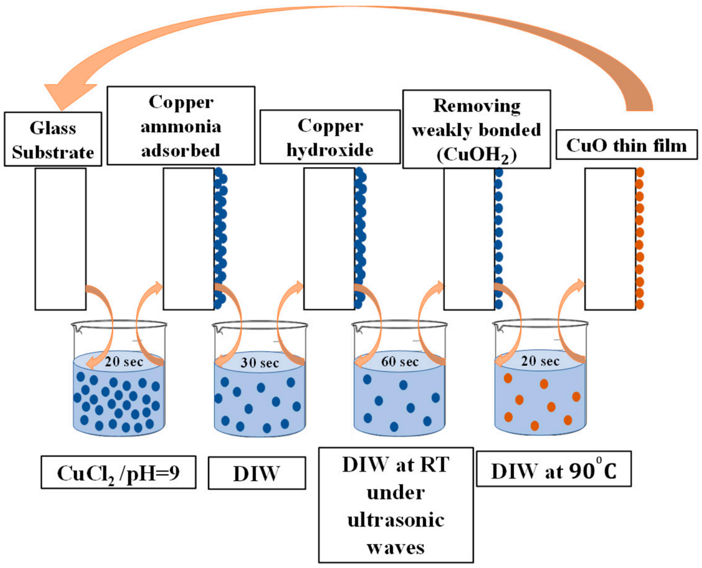

Figure S1. Schematic representation of the used modified SILAR technique to produce pure and doped CuO films.

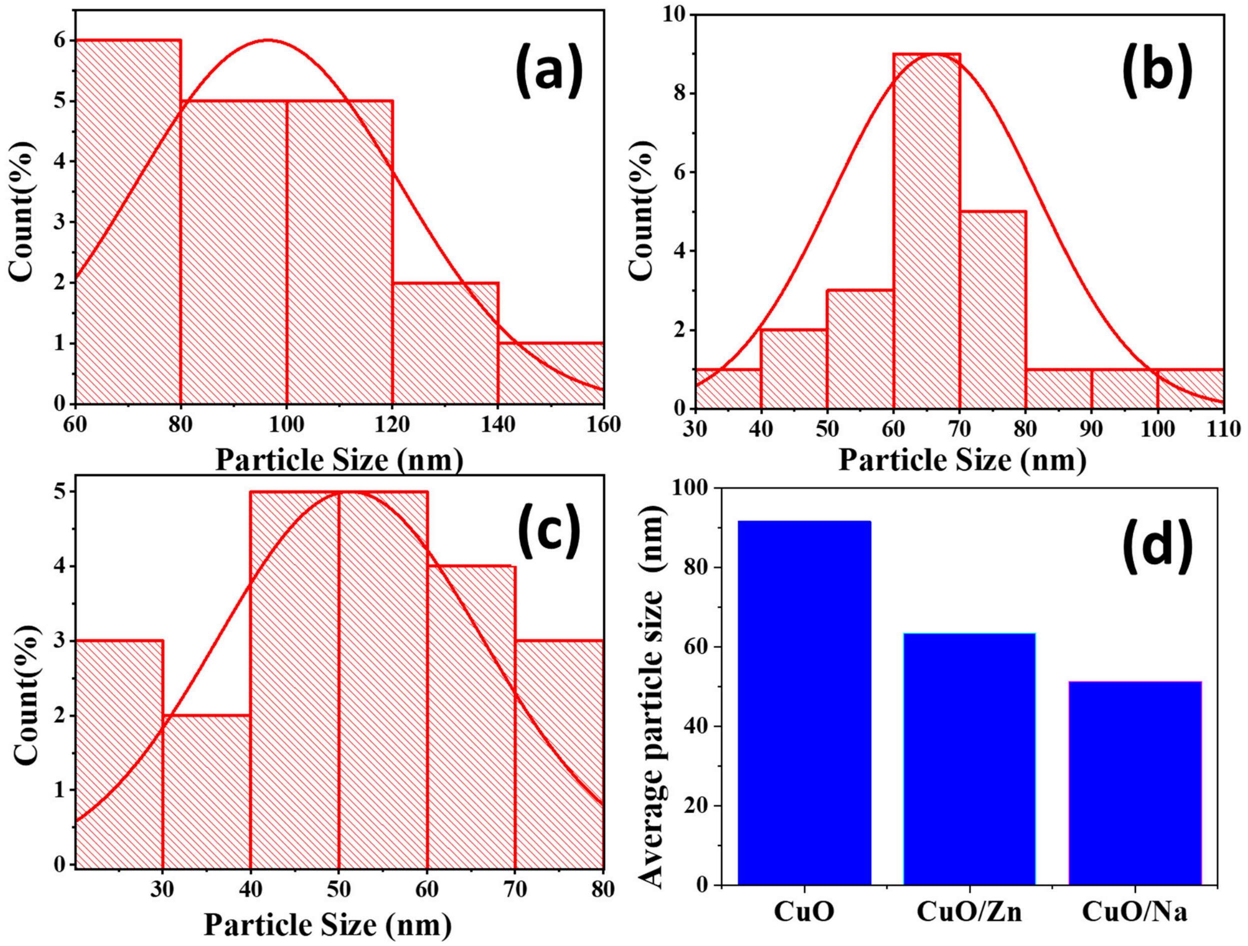

Figure S2. The particle size histogram for (a) CuO, (b) CuO/Zn, and (c) CuO/Na films.

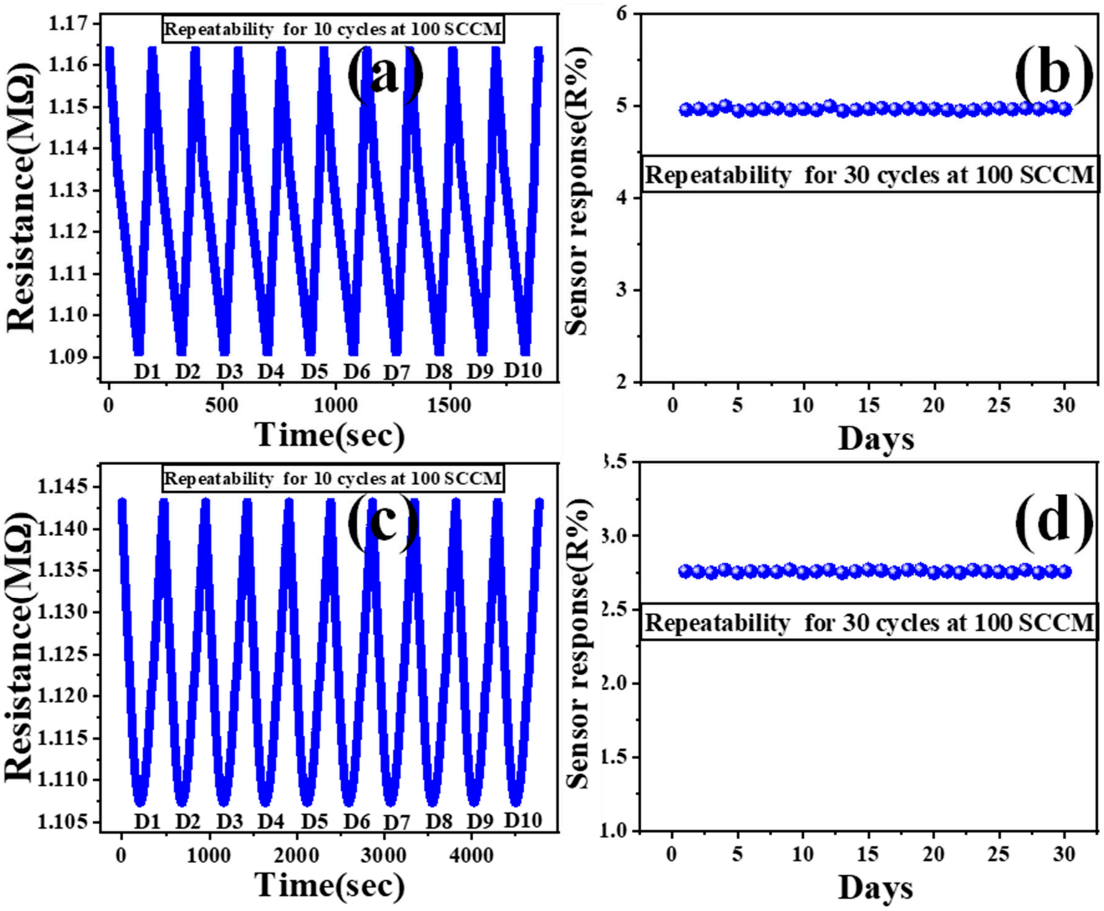

Figure S3. The repeatability of the CuO/Na when exposed to 100 SCCM flow rate of (a,b) CH_3OH and (c,d) NH_3 at room temperature in terms of (a,c) sensor resistance and (b,d) sensor response (R%).

Table S1. The oxidation state, ionic radius, and electronegativity for Cu, Zn, and Na.

	Oxidation state	Ionic radius	Electronegativity
Cu	+ 2	Cu^{2+} (0.072 nm)	1.90
Zn	+ 2	Zn^{2+} (0.074 nm)	1.65
Na	+ 1	Na^{+} (0.102 nm)	0.93
